# Paediatric Emergency Nurses’ Perception of Medication Errors: A Qualitative Study

**DOI:** 10.3390/nursrep14040223

**Published:** 2024-10-17

**Authors:** Blanca Collado-González, Ignacio Fernández-López, Valentina Urtubia-Herrera, Ana María Palmar-Santos, Eva García-Perea, María Victoria Navarta-Sánchez

**Affiliations:** 1 Hospital General Universitario Gregorio Marañón, Hospital Universitario de la Princesa, 28007 Madrid, Spain; blanca.collado@estudiante.uam.es; 2 Nursing Department, Faculty of Medicine, Universidad Autónoma de Madrid, 28029 Madrid, Spain; valentina.urtubia@estudiante.uam.es (V.U.-H.); eva.garcia@uam.es (E.G.-P.); maria.navarta@uam.es (M.V.N.-S.); 3 Buckinghamshire Health NHS Foundation Trust, Stoke Mandeville Hospital Accident & Emergency Departament, Aylesbury HP21 8AL, UK; ignacio.fernandez@nhs.net

**Keywords:** medication errors, nurses, paediatrics, emergencies, qualitative research

## Abstract

Patient safety is fundamental to healthcare. Adverse events, particularly medication errors, cause harm to patients, especially the paediatric population in the emergency department. Aim: To explore paediatric emergency nurses’ perceptions of medication administration errors. Method: A qualitative, ethnomethodological, descriptive study. The participants were nurses working in the paediatric emergency department. Data were collected through in-depth individual interviews with paediatric emergency nurses. The study excluded nurses employed for less than six months. Ten individual interviews were carried out. All interviews were face-to-face and audio-recorded with the participant’s consent. Interviews took between 52 min and 1 h 25 min. A questions guide was followed during the interviews. The analysis of the data was carried out according to the scheme proposed by Taylor and Bogdan. Results: The participants’ discourse revealed three main categories: Safety culture*,* transmitted by supervisors and safety groups. Teamwork*,* with good communication and a positive relationship. Error management, the lack of formal support and negative feelings despite an understanding of the multifactorial nature of errors. The study identifies several challenges in the healthcare system. Emphasis was placed on the perception of errors in terms of patient harm, while near misses or dose delays or omissions are not treated as errors. Conclusions: Although institutions have implemented safety culture strategies, nurses have not fully embraced them. There is a need to promote a positive safety culture and a safe working environment that encourages communication within the team. The hospital should provide training in safe management and patient safety and develop effective protocols. This study was not registered.

## 1. Introduction

Patient safety is a fundamental aspect of care [1]. Adverse events (AEs) cause great harm and damage to patients who suffer them. As healthcare-associated harm is considered a severe public health problem, strategies and practices are proposed to prevent and reduce the associated damage [1]. According to the World Health Organization (WHO), medication errors (ME) are the leading AE causing avoidable injuries and patient harm, with significant economic and social repercussions [2].

In comparison with the care of adult patients, MEs in the paediatric population are more frequent and present different characteristics. The precise calculation of doses, the narrow therapeutic margin, and the dilution of the drugs favour the possibility of making errors, both in the prescription and the administration process [3,4]. 

Although emergency situations in the paediatric population are less frequent than in adults, AEs nevertheless occur during the care of both populations [5].

### Background 

The prevalence of ME in the paediatric patient is not clearly established in the literature. According to the systematic review by Gates et al. [6] such errors in emergency departments range from 0.9 to 51.1 per 100 admissions in emergency departments using paper medication charts, decreasing to 15.1 (10.3–21.6) in those using a computerised system.

A Spanish study found that 12.3% of paediatric emergency department (PED) patients experienced an adverse event (AE), with 57.1% identified in later check-ups. Of these cases, 35.7% were medication-related [7].

The WHO in its “Global Patient Safety Action Plan 2021–2030: towards eliminating avoidable harm in health care” [1] stresses the need for a paradigm shift in the conception of error, avoiding blaming the professional. This change implies understanding error as a conjunction of factors, such as alterations in the functioning of systems, communication, organisational culture, rules and policies, in addition to purely human factors [1]. This approach to error could stimulate voluntary reporting of AEs and MEs, which is one of the main strategies to understand and prevent them. However, as Woo and Avery [8] suggest, voluntary reporting of AEs depends on personal factors, professionals’ perceptions and feelings, as well as the perceived institutional support.

However, to this date, no studies have been conducted to understand professionals’ perceptions of MEs and AEs.

This study is designed to understand the factors linked to the reporting of MEs, the experiences they generate in nurses, and strategies to ensure patient safety in the critical emergency setting and in the vulnerable paediatric population. Therefore, the primary objective of this study was to understand paediatric emergency nurses’ perception of ME. The secondary objectives were the following: to identify the emotions, thoughts, and behaviours generated after an ME; to explore the concept of safety during medication administration; and to describe and identify patient safety strategies resulting from MEs.

## 2. Materials and Methods

### 2.1. Design

A qualitative descriptive study with an ethnomethodology perspective following the method was described by Garfinkel [9]. This methodology was chosen owing to the complexity of the topic and the scarcity of previous studies, endeavouring to uncover nurses’ individual motivations, feelings, and meanings regarding their safety culture activities.

Ethnomethodology identifies activities and events with a focus on social action, subjectivity, and linguistic communication.

This approach seeks to gain in-depth knowledge of what happens in daily situations through paediatric emergency department nursing staff’s first-hand experiences [10].

### 2.2. Study Setting and Participants (Inclusion and Exclusion Criteria)

The study was conducted in the Community of Madrid (Spain), at level 2 and 3 hospitals and a paediatric hospital. The participants were nurses working in the paediatric emergency department (PED) of those hospitals.

Inclusion criteria consisted of more than 6 months of working experience in PED, excluding all professionals from the lead researcher’s work centre.

### 2.3. Data Collection

The main investigator (BCG) carried out ten individual interviews between March and July 2022 at the location agreed with the participant. All interviews were face-to-face and audio-recorded with the participant’s consent. Interviews took between 52 min and 1 h 25 min (on average 66 min).

The interviews were transcribed and assigned a code to eliminate participants’ personal or demographic data.

A question guide was followed during the interviews (Table 1). The question script was developed by the entire research team. The existing literature was reviewed, focusing on key questions related to the study objectives to develop the preliminary interview guide. After development of the questions, this interview guide was presented to two nurses with extensive experience in paediatric emergency care for review and feedback to ensure clarity and relevance and a final interview guide was obtained.

The questions were organised into five categories: safety barriers, the process of error, perception of support, personal impact, and strategies and solutions to error.

Access to participants was through the PED supervisors. The researchers ensured that there was no prior personal relationship with the participants. All participants who were informed agreed to participate. Only one participant was unable to attend the interview and withdrew.

### 2.4. Data Analysis

For data analysis we used the method proposed by Taylor and Bogdan [11]. This is a dynamic process, returning to previous steps as it unfolds. In the first phase of discovery, a literal transcription of the interviews was made, a careful reading of which extracted the first ideas. Annotations were made and compared to the field notes. Interviews were transcribed immediately after they were conducted.

In addition, the researcher used the field diary in each interview, which has been very useful in describing situations that were taking place, which did not belong to the verbal language category.

Subsequently, the encoding process began creating the initial categories and labels.

From these data, emerging themes were extracted, understanding the context in which they were said, the feelings that emerged during the interview, as well as the local and professional language used.

The search for typologies or classification schemes helps us to search for and develop theoretical propositions and concepts that allow us to describe the process and interpret it.

This process by which concepts are developed is intuitive, and to carry it out, we first searched the data collected for words or phrases from the informant that capture the meaning of what they say or do. Subsequently, the research team has compared the statements and actions to come up with a concept that unifies them and allows us to find the similarities between them.

In the last phase, the work previously carried out during the review of the current state of the subject was combined with the search for the perspectives, meanings, and social definitions that our participants give to each event, in order to arrive at the interpretation of the data collected. In order to do so, the data were relativised and reconstructed in the light of the context and the phenomenon under study.

### 2.5. Rigour and Reflexivity

To ensure the quality and acceptability of the research in this scientific field, we followed the criteria developed by Guba and Lincoln [12].

Confirmability was provided through detailed information on the steps carried out during the research, as well as the context of study and data collection.

Categories and subcategories were verified by all the team members, using quotes from participants and ensuring the accurate presentation and interpretation of participants’ experiences.

Transferability of the results was achieved thanks to a purposive sampling of participants to represent the study phenomenon in a heterogeneous range of settings (different work experience in PED services and different professional backgrounds). This wide variety of experiences provides a global vision of the study target and could further the understanding of similar contexts.

Credibility implies the need for reflexivity in researchers’ critical thinking regarding three aspects: self-reflexivity (own perspective) in the choice of theoretical perspectives and methodological design, reflexivity as a constant attitude justifying each step in the dynamic and flexible research process, and inter-subjectivity and two-way influence between researcher and participants. Transparency and identification of the researcher’s subjectivity as a research tool and its limitations assist the rigorousness and credibility of the process and its results.

Once the analysis was performed, the themes were worked out and the procedures of triangulation of fields and researchers were used. For triangulation, two paediatric emergency professionals, who were not involved in the study, were asked to review the data obtained. Regular meetings were held within the research team to discuss the results. In addition, data analysis was performed in the same way by the investigators with regular meetings [13].

To optimise rigour and transparency, this research work followed the consolidated criteria for reporting qualitative research COREQ [14] (Supplementary Materials, Table S1).

## 3. Results

Ten PED nurses at different hospitals participated in this study (Table 2). All nurses were female. Each participant was assigned a code suppressing hospital details to ensuring participants’ anonymity.

Sample size was considered adequate as it generated sufficient informative power in response to the objectives set. Data saturation was achieved. This was reflected in the fact that a wide variety of descriptions were obtained in the interviews and that no new ideas or new information were identified regarding the explored phenomenon [15].

**Figure 1:**
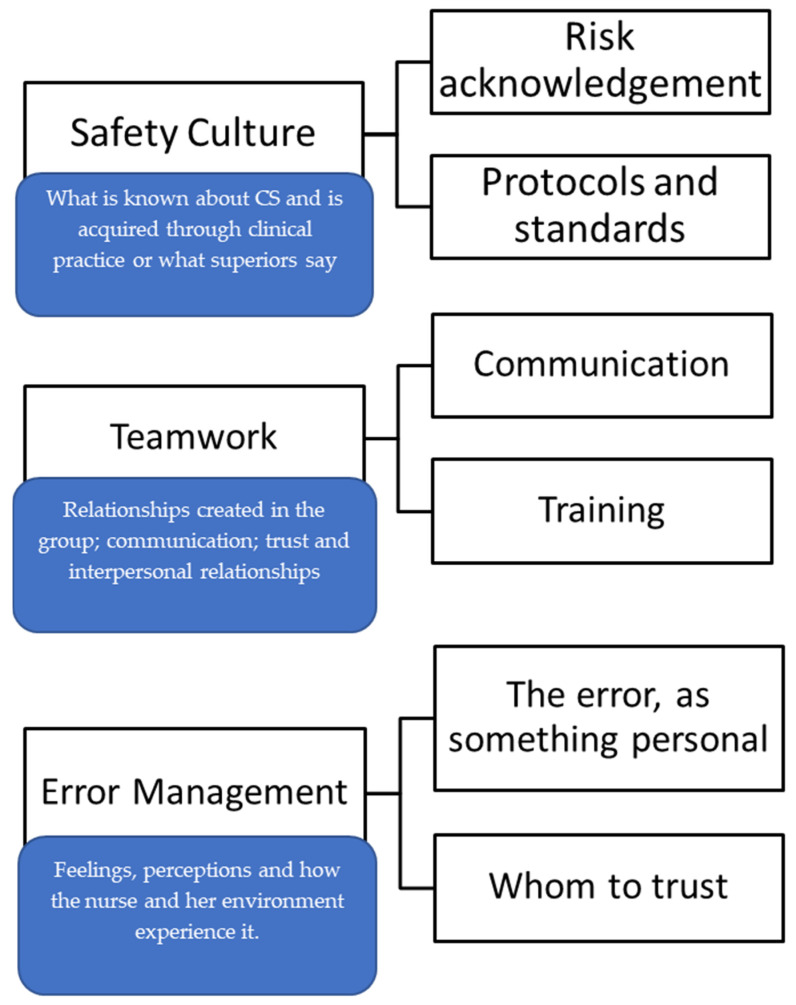
Categories and subcategories of the results on the perception of nurses and MEs.

From the data analysis we extracted three main categories: (1) Safety culture, (2) Teamwork, (3) Error management, which are explained below. These categories are represented in Figure 1 with their respective subcategories.

### 3.1. Safety Culture

The nurses refer to current pathways and protocols, and their own knowledge in relation to patient safety. They report that this knowledge is transmitted through different channels: the nursing supervisor, corporate mail, and sessions organised through the multidisciplinary groups set up to enhance and strengthen patient safety within the hospital.

The nurses report that the safety groups set up in the hospitals are a significant approach to inculcating the safety culture, and patient safety leads nurses’ work in these groups. Despite acknowledging the paucity of safety culture, nurses are of the opinion that these groups take important steps to instil a stronger safety culture. Likewise, these groups are responsible for reviewing and managing health incidents, including medication errors, among others.

“*It is true that here it is a bit like what we said in recent meetings that there is no culture of patient safety as such, we need a bit of a push to see what can be done, but well, there we are….*”

(E6)

Patient safety-led nurses are more proactive towards, and promote, changes. These same nurses state that involvement is personal, so they have no protected time within their working hours to carry out patient safety-related tasks.

“*It would be ideal was different, obviously, like those who are tutoring students who have assigned hours, so that they can do things with the students and for work, you know? The figure of the person in charge of security, no. It is true that it is a very good figure, but they need to be self-motivated.*”

(E9)

#### 3.1.1. Risk Acknowledgement

In the safety culture, nurses recognise that acknowledging actual risks is a key element in preventing ME. The nurses point out the most prominent risks: dosage calculation, interruptions, the wide variability of patients and professionals, as well as patient flow management and the stress induced in relatives, as the most significant risks.

“*Calculating dosage […] you receive the prescription in micrograms, but the medication is in milligrams, and you have to calculate it correctly if they tell you in mg and you have to administer it in ml and you have to dilute it or not dilute it, calculate it correctly and dilute it correctly and when administering it because if you use infusion equipment you have to know how long you have to administer it, […] if you have to administer it in 15–20 min or a continuous infusion you have to calculate it correctly.*”

(E5)

The PED environment makes it difficult to perform procedures established in other hospital units. The nurses contend that the rush in their daily work pressures them to override the times established in the protocols, which they understand as a safety breach.

“*These verbal orders are only supposed to be for the resuscitation area; what happens is, at the end of this is an emergency department, when there is a high volume of children, weekends, if you want to speed it up… it take long for them to prescribe it… they have seen two patients, and they are prescribing what is for the other one, for the other one what is for the previous one […] there is no time, right? The doctor takes longer to prescribe it.*”

(E9)

#### 3.1.2. Protocols and Standards

The nurses acknowledge that the institution develops protocols and regulations to ensure patient safety but point out that they are difficult to comply with in daily practice. They indicate that these protocols do not take into account the specific characteristics of paediatric emergencies and are not adjusted to the actual work routine. They also mention the need for greater operability and to manage patient care through patient flow and the prioritisation of needs at each moment.

“*You know what I mean, my impression of the institutions is they write protocols for patient protection or safety on paper nicely, but what actually happens in the ward is they are playing in two completely different leagues and have little relation to each other.*”

(E1)

In order to ensure compliance with these or the new protocols, the nurses consider that an authority figure such as the supervisor should watch over compliance. The patient safety specialist nurses do not accept this task.

“*I think the supervisor should be responsible for ensuring that her service complies. So, I think she should be the one, initially, when new rules come out, to be vigilant and somehow to check if people comply and at some point, it will become automatic. But that first time, maybe for a few months or even a year, I think they should be aware and, from time to time, go into the pits to see if they are being put in place or if they don’t. I think it should be her.*”

(E8)

The nurses admit to being familiar with the formal incident-reporting system, either through the nursing supervisor or through another colleague. However, they also admit to not completing it due to the difficulty it entails, the time spent on it, and the lack of knowledge of the objectives pursued by the system. They believe its ultimate purpose is to serve as a reminder and warn the individual.

“*I do not really know where that goes… it’s important to report it, especially why…? what’s the point if you do not put a name? If you do it anonymously… now I do not remember very well… you put the name of the person who did it? is it all anonymous? then I wonder what is the point…*”

(E4)

### 3.2. Teamwork

Teamwork is considered a key element to prevent ME and create strategies at individual and group levels. To this end, participants state that trust among team members is essential, regardless of professional category, along with interdisciplinary communication.

“*Because, of course, something I do not know, I do not dare to give initially if it makes me doubt I ask, you will ask the old ones.*”

(E9)

The nurses attest that the induction process for new staff members is the crucial moment for transmitting the safety culture, rules, and protocols, but relate that this is performed informally and is dependent on the goodwill of each professional. Participants state that as there is no specific protocol for this; safety strategies are acquired informally. Most senior professionals are increasingly reluctant to train new staff due to the high turnover. Despite this, they recognise that, in this regard, they do supervise the work of more junior colleagues in an unregulated manner.

“*There is no induction because an induction as such would have to be, it must be organised. Furthermore, there should be staff able to take that people. At some point, there was some kind of course, but well, for people, when you know that people arrive in the summer, new people arrive in bunches, but when are hiring every day and changing services, it’s impossible.*”

(E9)

#### 3.2.1. Communication

Communication is described as fundamental. Therefore, the lack of effective communication is recognised as a cause of ME or may increase the burden of nursing tasks, stressing the importance of respecting the necessary timeframes to prepare and administer drugs. Despite this, nursing staff accept interruptions while preparing medication.

“*Respect, I mean they respect you, that if I am or have been doing something, they do respect that and don’t interrupt me, if I’m loading medication they don’t come… someone doesn’t come and start talking to me or give me another medical order or…*”

(E3)

According to the nurses, communication of MEs and their possible solutions are informally transmitted to other professionals, who are thus not formally aware of the MEs that may have occurred in the service. They consider that holding team meetings to explain MEs and identify how and why they occur could be helpful in preventing them, as the failures share similar features. The nurses say that talking about ME would give professionals the confidence to ask questions and recognise personal limitations.

Nurses are usually aware about possible MEs, in their own or other departments, through colleagues’ comments, sometimes in the form of a personal critique of professional experience or training.

“*Initially, the minutes [of the safety meetings] only reach those of us who make up the safety group via email, and we informally comment on them, […] It is true if a more important conclusion is reached, the nurse supervisor sends us all a report, a note, via email.*”

(E8)

“*Yes, if you comment on whether this has happened, you have to be careful because depending on who has made the mistake, there is more gossiping or not.*”

(E5)

Communication with the family depends on the harm caused. The nurses involved in the error do not take responsibility for this task, which is delegated to one of the paediatricians who handle each case depending on the possible consequences for the patient. They admitted that the error is minimised vis-à-vis the family, even concealing it if there was no harm to the patient.

“*If it has happened and the patient has been harmed, yes, obviously, but if not… because, of course, it also depends on the family…*”

(E2)

#### 3.2.2. Training

Nurses view training as a key element in preventing ME. They demand continuous professional development training provided by the hospital.

“*For me, training in the emergency department is really vital, for me it is the panacea. What I see is the grade of patient safety mistakes decreases proportionally with the time you have been in the department because you know the drugs, you know the routes of administration, you know the doses.*”

(E1)

Younger nurses emphasise the lack of specialisation in nursing and the abrupt incorporation into a specialised and complex service, such as paediatric emergencies, without tutoring or specific knowledge.

“*We are not specialised in anything, and so we are good for everything, which may have some pros, but it also has many more cons because in the end, you are here at the age of 22 […] but I was sent here without any training, or care…*”

(E2)

Nurses underline the need to learn from AEs as a team without pointing fingers or blaming, creating strategies and barriers. They consider that most errors are due to common failures, stressing that talking about this will encourage a culture of safety and reporting.

“*Do not understand them as your own, try to understand it is something that happens a lot and that in the end, it is not that you make a mistake; it is that the system fails when it is something that happens a lot. It is not that you make a mistake, you individually as a person, nothing more. […] open your mind and try to focus on the objective, that it really is, that it is verbalise it and talk more about the subject so that it is not so taboo, and really see that the greatest good that we are going to achieve if this is done is that there will be fewer errors for the patient.*”

(E8)

### 3.3. Error Management

The nurses interviewed are aware of the multifactorial nature of ME. They reported that it can occur at any stage of the process, including system failures that ultimately allow the error to occur and sometimes reach the patient.

“*Because in the end, an error is a series of circumstances that add up and you’ve got it. That’s how it is. Because no, it’s not, it’s not you. It is rarely only a series of purely personal circumstances, but of patient’s circumstances, the situation of the service at that moment, staff, of things that come together…*”

(E9)

The nurses understand and are aware of mistakes, and therefore, MEs are part of the inherent nature of human beings. They express their desire not to make any, or in the event that they do happen, to minimise the possible consequences for the patient.

“*It is true that the fewer mistakes you make, the better, but I believe that zero error is impossible because we are human and… you are always going to make a mistake, and it better not be something serious.*”

(E3)

How an error is perceived differs depending on the harm caused. Consequently, there are unrecognised MEs. The perception is that a ME is administering more doses or giving them to the wrong patient. Missing a dose, a system failure or a recording error are not considered as MEs. Similarly, near misses are not counted as errors, as they do not actually occur and do not reach the patient in the end and are considered as part of the daily job routine.

“*I have not had medication errors as such, but perhaps I had other errors because when I worked in XXX I once left an infuser that did not beep and did not pump; I left an infuser with a closed morphine infusion, so that was detected because after many hours and one rescue dose after another and child had a huge pain spike that did not make any sense to us.*”

(E1)

Error management also differs depending on whether or not any damage is caused to the patient or on the measures taken. Thus, for ME with harm, nursing supervision is responsible for collecting more data on the incident, but if the event caused no harm, it is for the safety leads.

“*Well, when there is a consequence of this kind at the medical level and all that, normally yes, the supervision, if not, normally we are the ones who inform ourselves if nothing serious has happened….*”

(E6)

#### 3.3.1. The Error, as Something Personal

The participants conveyed feelings of guilt, fear and failure after committing an ME or being involved in one.

“*Yes, I see a sense of failure, and the colleague made it had a tremendously bad time.*”

(E9)

They also point to a loss of self-confidence and in their peers, feeling ashamed to recognise the failure.

“*I don’t know, to confirm I’m doing it right, because I panicked, maybe it was my experience, […] you have to be very careful and that it’s your responsibility.*”

(E10)

Nurses who have experienced an error at close quarters report different coping strategies in the aftermath and admit having become obsessive, repeating confirmation mechanisms.

“*I became obsessive with the clamps, used to check clamps even three times before carry on, think based on own´s personal experience, we developed some behaviours to continue practising our profession.*”

(E1)

These responses are amplified in terms of possible harm to the patient, accentuated by the existing blame culture in which the professional who makes the mistake continues to be pointed out individually. Nurses would even reach the point of seeking a change in profession in the event of serious harm to the patient.

“*I don’t want it to happen again, so it also depends a bit on the consequence of the medication error, how fatal the consequence is… Obviously, there is nothing to minimise the feeling you may have. Because if there is a fatal consequence, there are several deaths, one of them is the nurse who administered the medication.*”

(E9)

Others analysed the ME, looking for someone else to share the blame, or played down its importance claiming that no severe consequences ensued, or only a few, considering the length of their professional experience.

“*Because, of course, in the end, it might burden her too, well, not maybe, it will burden her, because in the end, this is a hospital, and there are the porters, the pharmacy, the emergency room, the nurses, the doctors, the assistants, the porters, OK? So it will burden everyone because this error burdens everyone, not just the nurses.*”

(E2)

Patient safety lead nurses are aware that the blame culture exists. They claim to be working to advance error management and analysis, avoiding blaming and looking for the overall failures in the system.

“*In general, I think each time we are managing to go more and more to the root of the problem, regardless of who made it.*”

(E9)

#### 3.3.2. Whom to Trust

Nurses involved in an ME stated that they sought support from people they trust, such as friends, family or other colleagues, and found it on an informal basis.

“*I mean, I’m not going to proclaim that I screwed up… with whoever was on the shift. Yes, of course, with colleagues who were not working on that shift but who are friends. I feel bad, and I don’t know what to say. Like, hey, I screwed up, look what happened.*”

(E5)

At the working centres hosting this study, the response from managers was erratic, causing staff to feel alternatively supported or attacked, with no regulated and structured professional support from the hospital.

“*The thing is that when the supervisor caught me by surprise, I didn’t say anything because I couldn’t say anything. I was in shock, and I was like… why are you telling me off? I was crying inside, I was saying what have I done?*”

(E2)

## 4. Discussion

This research is focused on learning and understanding nurses’ perceptions of ME. For nurses, patient safety is paramount, and they are aware of the existing risks. Despite this, the safety culture is not ingrained; there is no formal training on the subject, which generates feelings of guilt and anxiety after an ME.

Incidents occurring during clinical practice, in a non-negligent or malicious manner, are not exceptional, and have consequences for the professionals involved. Wu et al. [16] described negative psychological and emotional consequences on professionals who made an error. The occurrence of negative feelings found in our research affecting professionals indirectly involved in an error is consistent with previous studies, such as that of Delacroix et al. [17]. The European Network of Researchers Working with Second Victims [18] identified not only professionals who made an error, but also all the professionals indirectly involved in the event.

These feelings of anger, frustration, loss of confidence in themselves, loss of colleagues’ and patients’ confidence in their ability to work, become amplified if the damage caused to the patient is severe. These findings in our research match with previous studies proving how these feelings vary depending on the patient harm caused [17,19,20]. As in the literature review, near misses are not considered MEs by the professionals interviewed. For example, errors related to delayed administration are not reported [21,22].

The feelings generated by ME, as well as the fear of the institution’s possible response and being singled out and personally reprimanded by their own colleagues, is one of the reasons we found for the lack of formal AE notification. In the Spanish context, under-reporting is a prominent fact, as already noted in previous studies such as that of Granel et al. [23], in which they emphasise nurses’ failure to report AEs. The study by Dirik et al. [19] in Turkey highlights that 50% would not report an error, with a higher reporting rate for AEs with severe repercussions for the patient. Under-reporting may be reinforced by the current reporting systems, which professionals claim are non-practical. The same finding has occurred in previous studies [22,24]. In contrast, a Jordanian study on nurses found that the majority of their sample would report MEs [25]. Barriers to under-reporting include loss of trust from both peers and management, as revealed in our study. However, our research did not find fear of financial repercussions, a fact reported by previous international studies [24,25] that may be related to the context in which these studies were conducted.

In Spain, Ferrús et al. [26] stress the need to seek formal channels for reporting AEs and their solutions to avoid giving rise to rumours. In our work, in this sense, we found a lack of formal and non-punitive management of these AEs, with participants stressing the need to create formal channels to avoid rumours and misinformation. These improvements in management, with healthcare institutions providing resources and specific programmes to support secondary victims, could benefit not only the professional involved but the whole organisation, allowing for learning from the error, and promoting a proactive safety culture [27].

This lack of support was noticed in previous Spanish and international research [17,26], demonstrating that the way managers support their staff can minimise the secondary victim phenomenon [20].

International institutions advocate the promotion of quality and patient safety through undergraduate university training [28]. In Spain, the creation of functional risk management units, and hence, the emergence of the figure of patient safety lead, could help to change and strengthen the safety culture in healthcare centres [29]. In our study, patient safety-led nurses were more proactive and aware of patient safety. Mora et al. [30] already pointed out that this leadership position empowered and encouraged changes in safety and quality of care. Our research finds differences among these professionals´ opinions as tools for change. However, their roles are not defined in all the cases studied, and it is necessary to use more time to achieve a completed professional development and recognition.

The participants in our study, in line with previous studies, are aware that the causes that promote or encourage ME or AE are multifactorial. Among these, they highlighted interruptions [31,32], noise, and the lack of proper physical space to prepare medication [21,32,33], as well as patient rotation, the risks inherent to neonates or infants, underscoring the most critical moments of drug administration, such as the calculation of doses and dilutions. This is consistent with research conducted in the paediatric setting and in the emergency department, despite which they do not have formal strategies to prevent them [34].

Using the “Right five framework”, promoted by the World Health Organisation, is less effective in paediatric emergency departments, as we found in our research, and as other authors point out [35,36]. These studies showed that administration cannot be framed within a rigid system like the right five, because despite being a routine process, it is also a linear process [35,36]. Similarly, established safety protocols are not fully adhered to, which is not new and has already been confirmed in other research. Vaismoradi et al. [37] in their systematic review, found that nurses’ adherence to safety protocols is not complete. This could be due to external factors, such as interruptions or administration by parents without professional supervision, that do not comply with the right five framework in the administration of drugs.

Training in safety culture and continuous training to prevent MEs and AEs are demanded by the study participants, both newly qualified and experienced nurses. This improvement strategy is recommended by different authors [32,38,39].

### 4.1. Strengths and Limitations

This study provides relevant information on the daily work in paediatric emergency departments and is the first study with these characteristics carried out in Spain. It explores the perception of nursing staff within a specific microsystem, such as the paediatric emergency department, allowing us to better understand its organisation and professionals’ perception of EM. The diversity of hospitals and their organisation makes understanding easier and facilitates the study in other settings.

As limitations, we encountered the impossibility of capturing male profiles in the sample to explore whether this phenomenon has a gender-related construction. The fact that the vast majority of nurses are women has conditioned access to this aspect, but it would be interesting to include male profiles in future studies in case there could be a gender bias in the results. The results presented are based on a limited sample size. Nevertheless, the findings encompass a diverse range of hospital and nursing characteristics, thereby facilitating extrapolation. In addition, we have reached theoretical data saturation. No new insights have emerged.

### 4.2. Recommendations for Further Research

Knowing and understanding how these professionals manage AEs in the emergency department can help to understand the subsequent repercussions on the professional involved, as well as the repercussions on the team. In the same way, managers and middle management would seem responsible for promoting changes and instilling a fair and non-punitive safety culture. It is therefore necessary to understand how changes are made and how this information is transmitted to workers.

A possible element in promoting a change toward a proactive and non-punitive safety culture is the specialisation of nurses in patient safety. This suggests the need for further research into understanding the impact of nursing on the safety culture among healthcare professionals.

Likewise, it would be desirable to carry out comprehensive studies exploring the concept of safety culture among all PED professionals, as a work team, encompassing the context and the characteristics of the work environment.

Implications for policy and practice:

This study highlights the disparity, within the public health system, of compliance with protocols and safe practice. It is necessary to establish strategies common to all hospitals, encouraging teamwork through multidisciplinary meetings to enhance communication between professionals and patient safety units.

Nurses are key to preventing MEs, and their involvement is essential in drafting and implementing protocols. Nurses should be given roles of responsibility and leadership and, therefore, greater knowledge, commitment, and sensitivity regarding safety and quality of care.

## 5. Conclusions

This study aimed to investigate the perceptions of nurses in relation to medication safety in the PED environment.

Nurses dedicate a large part of their working day to administering medication, with patient safety being a top priority. Therefore, their discourse provides valuable insights into safety culture practices, attitudes, and thoughts. This study emphasises the importance of comprehensively understanding the concept of safety culture as perceived by nurses, and their attitudes towards risk in their professional practice.

Protocols and strategies have been implemented by institutions to promote quality care and patient safety. However, the safety culture is not firmly established in practice units, and compliance with protocols is inconsistent, with only those deemed practical and feasible being applied.

There is no formal safety culture or patient safety training provided within the institutions. This results in professionals having to acquire knowledge informally, which is not conducive to effective learning.

The absence of formal channels and specific training in safety culture and patient safety perpetuates a culture of blame, leading to feelings of guilt, frustration, and anxiety. 

Training is highlighted as the most effective strategy to prevent ME, along with promoting teamwork and formal communication.

This exploratory study demonstrates that the safety-related behaviours of professionals are culturally and contextually determined, highlighting the need to comprehend this reality. Understanding each particular work setting is crucial to implementing effective processes and institutional dynamics that align with the context, patients, and professionals. The work environment is thus optimised for all parties involved.

## Figures and Tables

**Table 1:** Interview script.

Topics	Questions
Safety barriers	Which patient safety policies are implemented? Who makes those policies?
	What is your opinion about those safety policies?
	Do you feel any difficulties putting them in place? What is the team’s opinion? In your opinion, what is the most relevant aspect of safe clinical practice?
Making an error process	Why do you think ME happen? When is the most critical moment for treatment administration?
	Who are the most vulnerable patients? About an ME, could you tell me in your opinion what happened? If you were back in time, is there anything you could have done differently? Did you talk to your colleagues? How do you manage the situation with the patients? Is it always the same protocol? In case of a ME, do you inform the relatives? Who does it? Do you think relevant they know about it? What is your perception of other colleagues’ experiences? How is it felt among the team when a ME happens? What is your opinion about ME notification? Do you consider it useful to prevent errors? What is your guess about your colleagues’ ME perception? In your opinion, which factors contribute to a ME?
Support perception	What happens in the department when an ME occurs?
	In case an ME happens while you are on shift, how is an ME managed? Are they always managed in the same way? What it depends on?
	Do you do some training with the team after a serious ME happened? Do you think there is any factor that helps to minimise the consequences on the team?
	What is the response from the management team and the institution?
Personal repercussion of making a mistake	Did this incident have any repercussions on the rest of the shift? On the following days? Has an ME, either yours or by a colleague, changed the way you interact with the team, relatives, or patients? What did you feel when you realise you made a mistake?
	How would you feel if they implemented a new policy after you or a colleague made a mistake? Has any of these ME changed the way you work?
Solutions	What is your perception about safety measures taken in place, for example, a double check of medication? Do you think the current reporting system is useful and practical? Do you think there is a way to avoid ME or to get almost all ME to get reported? In your opinion, what is the most relevant aspect of the topic? If you could decide, would you make any changes? How do you feel when a new clinical safety measure is implemented?

**Table 2:** Participant’s characteristics.

Code	Age	Safety Lead	Years in Paediatric Emergency
E1	35	No	7 years
E2	23	No	20 months
E3	36	No	16 years
E4	55	No	17 months
E5	35	No	18 months
E6	39	Yes	16 years
E7	39	Yes	5 years
E8	35	Yes	6 years
E9	50	Yes	7 years
E10 *	27	No	3 years

* Paediatric specialist nurse via residency program.

## Data Availability

The original contributions presented in the study are included in the article, further inquiries can be directed to the corresponding author.

## Supplementary Materials

The following supporting information can be downloaded at: https://www.mdpi.com/article/10.3390/nursrep14040223/s1, Table S1: Consolidated criteria for reporting qualitative studies (COREQ): 32-item checklist

## Public Involvement Statement

No public involvement in any aspect of this research.

## Guidelines and Standards Statement

This manuscript was drafted against the Consolidated criteria for reporting qualitative research (COREQ) Tong, A.; Sainsbury, P.; Craig, J Consolidated criteria for reporting qualitative research (COREQ): A 32-item checklist for interviews and focus groups. *International journal for quality in health care* **2007**, *19*, 349–357, DOI 10.1093/intqhc/mzm042.
