# Supplementary material for: Paediatric Emergency Nurses’ Perception of Medication Errors: A Qualitative Study
Source: Nurs Rep. 2024 Oct 17;14(4):3069–83. doi: 10.3390/nursrep14040223 (PMC11503309; doi:10.3390/nursrep14040223)
Supplement: Supplementary file 1 [file nursrep-14-00223-s001.zip › nursrep-3203119-supplementary.pdf]

Table S1
Consolidated criteria for reporting qualitative studies (COREQ): 32-item checklist

Please indicate in which section each item has been reported in your manuscript. If you do not feel an item applies to your manuscript, please enter N/A.

For further information about the COREQ guidelines, please see Tong *et al.*, 2017: https://doi.org/10.1093/intqhc/mzm042

No.	Item	Description	Section #
**Domain 1: Research team and reflexivity**			
Personal characteristics			
1.	Interviewer/facilitator	Which author/s conducted the interview or focus group?	BCG
2.	Credentials	What were the researcher's credentials? *E.g.PhD, MD*	BCG:RN; MSc; PhD student;<br>IFL: RN; MSC<br>VUH:RN; MSc; PhD student;<br>EGP:RN;MSc; PhD<br>VNS:RN;MSc; PhD<br>APS:RN;MSc; PhD
3.	Occupation	What was their occupation at the time of the study?	BCG:Nurse in a high complexity hospitals (HCHs Nurse<br>IFL: Advanced Clinical Practitioner MSc PGDip BScN(Hons) RN<br>VUH: PhD student<br>EGP: Academic<br>APS: Academic; VNS: Academic
4.	Gender	Was the researcher male or female?	BCG: Female<br>IFL: Male<br>VUH: Female<br>EGP: Female<br>VNS: Female<br>APS: Female
5.	Experience and training	What experience or training did the researcher have?	BCG, IFL, VUH: more than 4 years of experience<br>EGP, VNS & APS: Extensive experience
Relationship with participants			
6.	Relationship established	Was a relationship established prior to study commencement?	A person from outside the research team offered to participate in the research. Interested individuals were contacted via email to invite their participation.
7.	Participant knowledge of the interviewer	What did the participants know about the researcher? *E.g. Personal goals, reasons for doing the research*	Participants were informed in writing of the focus of the study, the role of the researcher in the study, the objectives, and the time allotted prior to the start of the research and interview.
8.	Interviewer characteristics	What characteristics were reported about the interviewer/facilitator? *E.g. Bias, assumptions, reasons and interests in the research topic*	BCG has been a pediatric emergency nurse for seven years. This experience may have influenced the interviews and analysis of the qualitative data. VUH, IFL, EGP, APS and MNS participated in the analysis.

**Domain 2: Study design**			
Theoretical framework			
9.	Methodological orientation and theory	What methodological orientation was stated to underpin the study? *E.g. grounded theory, discourse analysis, ethnography, phenomenology, content analysis*	The methodological orientation was ethnography (Garfinkel 2006) and the data analysis was according to the approach advocated by Taylor and Bogdan (2002).
Participant selection			
10.	Sampling	How were participants selected? *E.g. purposive, convenience, consecutive, snowball*	Purposive sampling.
11.	Method of approach	How were participants approached? *E.g. face-to-face, telephone, mail, email*	First, we initially contacted them by email. Once they confirmed their participation, we scheduled an appointment via telephone.
12.	Sample size	How many participants were in the study?	Ten
13.	Non-participation	How many people refused to participate or dropped out? What were the reasons for this?	One person refuses to participate because he doesn't have time within his working hours
Setting			
14.	Setting of data collection	Where was the data collected? *E.g. home, clinic, workplace*	Interviews were conducted face-to-face at the workplace nine of them, and one was at the participant´s home.
15.	Presence of non-participants	Was anyone else present besides the participants and researchers?	The interviews were conducted in private places, with only the researcher and the participant present.

16.	Description of sample	What are the important characteristics of the sample? *E.g. demographic data, date*	The sample consisted of ten nurses, all ten of whom were women. With different work experience in pediatric emergency care. The one with the least experience (17 months) had worked as a nurse for 34 years. Four nurses were safety referents and belonged to different hospitals with different characteristics.
Data collection			
17.	Interview guide	Were questions, prompts, guides provided by the authors? Was it pilot tested?	The guide was developed based on existing literature. It was reviewed by the research team and the ethics committee. However, participants were allowed to speak freely about their thoughts and feelings about the topic under discussion.
18.	Repeat interviews	Were repeat interviews carried out? If yes, how many?	No interviews were repeated
19.	Audio/visual recording	Did the research use audio or visual recording to collect the data?	We used audio recording
20.	Field notes	Were field notes made during and/or after the interview or focus group?	During and after the interview
21.	Duration	What was the duration of the interviews or focus group?	54min- 85min
22.	Data saturation	Was data saturation discussed?	Yes
23.	Transcripts returned	Were transcripts returned to participants for comment and/or correction?	No
**Domain 3: analysis and findings**			
Data analysis			
24.	Number of data coders	How many data coders coded the data?	Five
25.	Description of the coding tree	Did authors provide a description of the coding tree?	Yes
26.	Derivation of themes	Were themes identified in advance or derived from the data?	Themes were derived from the data following the approach of Taylor and Bogdan (2002)
27.	Software	What software, if applicable, was used to manage the data?	Excell
28.	Participant checking	Did participants provide feedback on the findings?	No
Reporting			
29.	Quotations presented	Were participant quotations presented to illustrate the themes / findings? Was each quotation identified? *E.g. Participant number*	Yes we have included quotations, however, participants were pseudonymized and not identified

30.	Data and findings consistent	Was there consistency between the data presented and the findings?	Yes
31.	Clarity of major themes	Were major themes clearly presented in the findings?	Yes
32.	Clarity of minor themes	Is there a description of diverse cases or discussion of minor themes?	Yes, this is presented within the major themes. (e.g. "Communication")

When submitting your manuscript via the online submission form, please upload the completed checklist as a Figure/supplementary file.

If you would like this checklist to be included alongside your article, we ask that you upload the completed checklist to an online repository and include the guideline type, name of the repository, DOI and license in the *Data availability* section of your manuscript.

Developed from: Allison Tong, Peter Sainsbury, Jonathan Craig, Consolidated criteria for reporting qualitative research (COREQ): a 32-item checklist for interviews and focus groups, International Journal for Quality in Health Care, Volume 19, Issue 6, December 2007, Pages 349–357, https://doi.org/10.1093/intqhc/mzm042
